# Efficiency Analysis and Comparison of Different Radon Progeny Measurement Methods

**DOI:** 10.1155/2013/269168

**Published:** 2013-12-05

**Authors:** Abdumomin Kadir, Lei Zhang, Qiuju Guo, Juncheng Liang

**Affiliations:** ^1^State Key Laboratory of Nuclear Physics and Technology, School of Physics, Peking University, Beijing 100871, China; ^2^Solid Dosimetric Detector and Method Laboratory, Beijing 102205, China; ^3^Ionizing Radiation and Medical Science, National Institute of Metrology, Beijing 100013, China

## Abstract

Radon exposure to the public contributes more than half of all the radiation doses caused by natural radiation; accurate measurement of radon progeny is quite essential for the dose evaluation of radon exposure in environment. For the purpose of establishing a radon progeny standard and controlling measurement quality of commercial devices, it is quite important to analyze the efficiency of different measurement methods and determine which would be the most appropriate for radon progeny measurements. Through theoretical analysis and experimental measurement, some commonly used measurement methods were compared in this study and the development trends of those methods were reviewed. Results show that for radon progeny measurement, the spectroscopic analysis method is better than the gross count method, while least-square calculation methods is better than traditional three-count or five-count method. Multiperiod counting of *α* plus *β* spectrum as well as using weighted least-square calculation method might be the best choice for accurate measurement on radon progeny in standard radon chamber when calibrating commercial radon progeny monitors.

## 1. Introduction

Radon is one of the most important sources of natural radiation. With the inhalation of short-lived radon progeny in ambient, it can cause internal exposure and might lead to lung cancer [1]. The measurement of radon progeny concentration is important for dose evaluation. Many measuring devices and many measurement methods have been developed in the last century. Moreover, a large number of commercial radon progeny monitors were developed and widely applied in environmental survey nowadays. For quality control on measurement, a large number of reference radon chambers [2–4] were built for establishing radon and radon progeny standard and for assessing the quality of those devices and instruments as well.

With the purpose of setting up Chinese primary radon standard, a 20 m^3^ radon chamber assembled with stainless steel has been built recently in National Institute of Metrology (NIM), China. It is equipped with temperature and humidity controlling system and could adjust ventilation. To realize the controlling of radon progeny, aerosol generator and aerosol measurement device such as Condensation Particle Counter (CPC, TSI Inc. model 3781) and Scanning Mobility Particle Sizes (SMPS, TSI Inc. model 3475) are also equipped with special design in aerosol entrance and sampling part. To assess the quality of radon progeny monitors, we are trying to find an accurate measurement method and build an accurate measuring device in order to build a reference standard of radon progeny.

In this paper, we try to analyze the efficiency of different measurement methods for radon progeny and review the history of those methods firstly. Then through theoretical analysis and experimental comparison under pure radon environment, we try to figure out which one is the most effective measurement method and what we should do to get result that is more accurate.

## 2. Basic Principles of Radon Progeny Measurement

Radon progeny concentration usually can be expressed either by their individual concentration or by their Potential Alpha Energy Concentration (PAEC) which is a linear combination of individual concentration, so radon progeny measurement can be divided into two kinds—individual progeny's concentration measurement and PAEC measurement. Radon (^222^Rn) progeny being interested here are ^218^Po (alpha emitter, T_1/2_ = 3.05 min), ^214^Pb (beta emitter, T_1/2_ = 26.8 min), ^214^Bi (beta emitter, T_1/2_ = 19.7 min), and ^214^Po (alpha emitter, T_1/2_ = 1.64 × 10^−4^ s). Because of the relatively short half-life of ^214^Po compared to its parent atom ^214^Bi, it is always assumed that radioactive equilibrium is established between them. Since we care more about the different radon progeny's concentrations, so in this paper, without exception, the radon progeny concentration means the concentration of individual radon progeny.

In order to get each progeny's concentration, measurement processes usually involve two steps—sampling and counting (or measurement). Sometimes those two processes could separate by time; sometimes those two processes could work at the same time. In sampling process, radon progeny in ambient air are collected on a filter. In counting process, the alpha or beta particles emitted from decayed radon progeny on the filter are recorded by detector. Using spectra analyzing technology, we can get either gross count or counting rate of different particles in different time intervals. Now the question is how to calculate the concentrations of different radon progeny using those multiperiod counts or counting rates.

Before extrapolating the relationship between time-interval counts and concentrations of radon progeny, we need to do some basic assumptions as follows: first, progeny concentrations remain constant during sampling period. Second, pump velocity, collection efficiency, and counting efficiency remain the same during measurement. Last, environmental parameters nearly have no influence on measuring process. The validity of the first assumption will be discussed later in this paper when making practical measurements in the field.

Based on those assumptions, we can easily establish the relationship between time-interval counts and concentrations of radon progeny. Taking into account the accumulation and the decay of different progeny during the sampling and counting period, using the ending point of sampling as the initial time of counting (*t*′ = 0), the time variation of the progeny atom's number on the filter can be expressed as follows [5]:

(1) $$\begin{array}{c} \frac{dN_{i}\left(t\right)}{dt}=C_{i}v+\lambda_{i-1}N_{i-1}\left(t\right)-\lambda_{i}N_{i}\left(t\right),\;\left(i=1,2,3\right), \end{array}$$

(2) $$\begin{array}{c} \frac{dN_{i}\left(t^{\prime }\right)}{dt^{\prime }}=\lambda_{i-1}N_{i-1}\left(t^{\prime }\right)-\lambda_{i}N_{i}\left(t^{\prime }\right),\;\left(i=1,2,3\right). \end{array}$$

Equation (1) is for the sampling process while (2) is for the decay process after sampling, where *v* is flow rate (lpm), *λ*_*i*_ is decay constant (*s* ^−1^), *C*_*i*_ is radon progeny's concentration (Bqm^−3^), *i* = 1,2, 3 stands for ^218^Po, ^214^Pb, and ^214^Bi separately. Taking initial condition Ni (*t* = 0) = 0 and Ni (*t*′ = 0) = Ni (*T*) (where *T* means sampling time), solving (1) and (2), we can calculate out the number of radon progeny atoms on the filter during sampling and counting period.

Solving (1) and substituting it into (2), considering the collection efficiency of filter, and counting efficiency of the detector, we can get the relationship between radon progeny's concentrations and gross count or counting rates in different time intervals. The expression of this relationship is as follows:

(3) $$\begin{array}{c} M_{j}=a_{j1}C_{1}+a_{j2}C_{2}+a_{j3}C_{3}, \end{array}$$

where *M*_*j*_ is total count or counting rate during *j*th time interval. *a*_*ji*_ is a constant that is determined by parameters such as sampling time, counting time intervals, decay constant, flow rate, and collection and detection efficiency.

Here we can see at least three measurement values should get for determination of three progeny's concentrations. That means *j* must be larger than three. If *M*_*j*_ is total alpha count of one interval, at least three counting intervals are needed, such as Thomas Three-Count Method [3]. While if *α* particles from ^218^Po and ^214^Po are differentiated, we need only two counting intervals to get three *M*_*j*_ for radon progeny calculation, such as the Kerr method [4]. Usually, more measurement data (more of *M*_*j*_) probably means more accurate estimation of radon progeny concentrations, but it also mean a calculation process that is more complex and might need longer measuring time such as Raabe and Wrenn least-square method [6]. Due to the possible change of radon progeny's concentrations in actual environment, we are trying to get more accurate measurement result in a shorter measuring time and then shorter total time. That is the aim of all our optimization of those measurement methods.

## 3. The Development of Radon Progeny Measurement Methods

In order to find a more accurate measurement method and try to optimize those methods, we first do a short review of some classical measurement methods of radon progeny. Through reviewing, we might find some interesting development trends.

The measurement methods of radon progeny have developed for more than sixty years. Tsivoglou et al. created a classical measurement method early in 1953 [7]. This is one of the earliest measurement methods, and it has been used widely ever since. The method records the total alpha counting rate at 5, 15, and 30 min after end of the 5 min sampling, and the three-radon progeny concentrations are calculated by resolved calculation. Due to its simplicity, this method was wildly used in the mine and commercial instruments.

Following Tsivoglou et al. method, Thomas pointed out that it would be more precise, by replacing counting rate at certain time with total counts in different interval. In 1972, he created the famous Thomas Three-Count Method [3]. This method records the total alpha counts in 2–5, 6–20 and 21–30 min after 5 min sampling. Following this thought, Nazaroff et al. [8] and Tian and Lu [9] proposed different counting intervals to optimize this method. Nowadays, Thomas Three-Count Method is still one of the classical measurement methods and widely used in radon progeny measurement.

Sometimes, three-interval counting rates are enough for radon progeny's concentration measurement. If more counting rates are given, the measurement results might be more precise. Following this thought, Raabe and Wrenn first applied least-square method to calculate the radon progeny's concentrations in 1969 [6]. This method records the alpha counting rates at every 3 min and uses the least-square fit for calculation. Numerical solution gives precise results through computer program. Recently, Mingli et al. did some optimization on this method and proposed a similar method that replaces the counting rate in Raabe and Wrenn Method with total alpha counts in 8 or 10 time intervals [10].

With the development of spectra analyzing technique, more information than total counts was recorded. The counts of different alpha particles were separately recorded. Using spectra analyzing technique, we could get the net counts of 6 MeV as well as 7.69 MeV alpha particle. Martz et al. first used alpha spectroscopy in radon progeny concentration measurement in 1969 [11]. Not like Tsivoglou et al. method, they recorded three alpha decay rates at only two different time intervals. Recording the counting rate of 6 MeV alpha and 7.69 MeV alpha at the first time interval as well as the counting rate of 7.69 MeV alpha at the second time interval, the progeny concentrations could be calculated through resolved calculation. Jonassen and Hayes replaced the three counting rates with three counts of different alpha particles in two intervals [12]. Kerr et al. made an optimization to former method and developed a procedure to measure Rn-222 as well as Rn-220 progeny using alpha spectroscopy [13].

Actually, radon progeny emits *β*, *γ* as well as *α* particles. Due to the quick development of gamma spectrometry and beta spectrum analysis technology, there appeared some methods like “*β* count” method [14, 15], “*α*-*γ* spectra” method [16], and “*α*-*β* count method” [17]. Although those methods are not widely used in commercial device, they give us a cue to improve our current method. That is, more information means results that are more precise. Rolle did a comparison of different radon progeny measurement methods and pointed out that simultaneous detection of *α* and *β* particles might improve the precision of radon progeny concentrations measurement greatly [18]. With the development of Passivated Implanted Planar Silicon (PIPS) detector and spectra analyzing technique, “*α*-*β* count method” might be more useful than “*α*-*γ* spectra” method, though the later method is nowadays used as a prime standard in PTB [19].

Through reviewing the development of radon progeny measurement method, we could easily find out the following trends. First, radon progeny measurement methods have went through a trend from measuring counting rate to time-interval count and then to alpha/beta spectra. Nowadays, *α* spectroscopic analysis method has become the dominant method, but with the adding of beta's information, the “*α*-*β* spectroscopic method” will achieve great development in the future. Second, the calculation method has gone through a development from resolved calculation to numerical calculation, such as from “three-integral method” to “weighted least square method.” Effective increment of the number of the measured value will surely improve the precision, so “least-square method” will certainly be used in the future more frequently. Combining simultaneous *α*/*β* spectral counting and least-square analyzing method, this will surely be a method of faster responding and higher precision, which will not only promote the measurement precision but also shorten the response time of radon progeny measurement.

## 4. Theoretical Analysis of Different Radon Progeny Measurement Methods

Due to the variability of radon concentration in environment, the ultimate goal of radon progeny measurement is to get more accurate progeny concentration in shorter time as could as possible. However, different measurement methods have different sampling and counting process as well as different data analysis method. Without a uniform quantity, it is hard for comparison. Therefore, we define *Efficiency Index*, EI = 1/(SD × TT), where SD is standard deviation (SD), which is a normalized value by the square root of the standard deviation of each radon products. A derivation of SD is given in the appendix of Rolle's paper [18], total time (TT) is the sum of sampling time and counting time. For the same total time, the smaller the SD, the higher the EI; if we need the same SD, the shorter the total time, the higher the EI. EI is a quite suitable value to illustrate the efficiency of different measurement method.

For comparing with the result in Rolle's paper, we used the same parameters in Rolle's paper [18]. The concentration of ^218^Po, ^214^Pb, and ^214^Bi is 341, 197, and 113 Bqm^−3^ in this environment, PAEC of ^222^Rn is 1 *μ*J/m^3^ with equilibrium factor *F* = 0.5. Assume collection efficiency is one under unit sampling velocity (1 lpm). The detection efficiency of *β* and *α* comes from ^218^Po is 0.005 and 0.25, *β* and *α*_1_, *α*_2_ from ^214^Bi/^214^Po is 0.15, 0.025 and 0.22, while *β* comes from ^214^Pb is 0.15. Thinking of different methods' processes, solve (1) and (2), and then calculate out *a*_*ji*_ in (3) combining all those parameters. We could figure out the EIs of different measurement methods. All those calculations were done through programs written in MATLAB. The calculation results are shown in Table 1.

For comparison with Rolle's result, the standard deviation of PAEC is also shown in this table. Our calculation results are nearly the same with Rolle's results. Different measurement methods' process is also given in Table 1, where the time initial is the start time of sampling. The bracket (–) means recording the counts in this time intervals. Those suspension points mean repeating count every 3 min. *α*_1,2_ means recording total *α* counts with no energy differentiation, *α*_1_ *α*_2_ means measuring *α* spectrum, and *α*_1_ *α*_2_ *β* means measuring *α* and *β* spectrum simultaneously. The following number in bracket, 8 or 10, means recording for 8 or 10 time intervals. For example, *α*_1_ *α*_2_ *β*(8) means we record alpha and beta spectrum simultaneously and separate 6.0 MeV- and 7.69 MeV-alpha from Po-218 and Po-214. We repeat 8 times each with 3 min interval.

From those results, we could easily find out that different methods have quite different efficiency. The spectrum methods are more efficient than total counting method by comparing Thomas and Nazaroff methods with Kerr method. Even using least-square calculation method, recording *α* spectrum will surely give more information and lead to a more efficiency result, such as comparing 4 and 5.

Comparing Kerr method with *α*_1_ *α*_2_(8) as well as *α*_1_ *α*_2_(10), we could see that though the efficiency is nearly the same, we could get more precise PAEC in short time. Ten time-interval method *α*_1_ *α*_2_(10) will give more precise PAEC than 8 time-interval method *α*_1_ *α*_2_(8). The least-square method will be better for radon progeny calculation in certain extent, though sometimes not for ^218^Po, as it will be shown below.

Comparing *α*_1_ *α*_2_ *β*(1) with Kerr method and comparing 7 with 4, we could easily find that the *αβ* spectrum method will greatly improve our nowadays measurement methods' efficiency. That means we could get more precise measurement result in shorter time.

## 5. Experimental Comparison of Different Radon Progeny Measurement Methods

For further validating the theoretical results above, experimental comparisons were carried out in the national radon chamber at NIM, with a radon concentration of nearly 7000 Bqm^−3^ and aerosol concentration nearly 10^5^ cm^−3^. Radon progeny is collected on filter by grab sampling method at a flow rate of 1 lpm. Two samples were collected at the same time, the first samples for 5 min and the second samples for 10 min. After 1 min decay, those samples are put into ORTEC Alpha-Duo spectrometer, which have two vacuum channels for measurement at the same time. The detection efficiency of alpha and beta is 0.370 and 0.275 through calibration separately. Measured *αβ* spectrum was saved automatically every one minute. Those spectrums were analyzed by program to get the net counts in those intervals given in Table 1. Through resolved or numerical calculation, radon progeny's concentrations are obtained. However, most of time we care more about ^218^Po concentration and Equilibrium Equivalent Concentration (EEC) than each radon progeny's concentrations. The result of radon progeny concentration is shown in Table 2.

Experiment comparison shows that the EECs of different measurement methods are nearly the same, except the Thomas method that has a difference less than 10%. Through comparing the SD of PAEC, we could also find that spectrum analysis method gives a smaller SD than total counting method and *αβ* spectrum method gives smaller SD than *α* spectrum method. In addition, 10 time-interval method seems better than 8 time-interval method. Experimental comparison and theoretical analysis agree very well.

However, the concentration of ^218^Po presents great dispersion between different methods compared to other two progeny's concentration as shown in Table 2. Especially the Kerr method gives a much higher Po-218 than other methods, such as alpha spectrum and alpha/beta spectrum methods. Then, which method is the best evaluation of Po-218 concentration?

By carefully analyzing those factors that affect the precision of ^218^Po concentration, we could find that the most important factor is the total count in the first time-interval, because ^218^Po has quite short half-life, 3.05 min. Therefore, the more counts from ^218^Po are recorded, the more precise the results of ^218^Po concentration might be. As showen in Table 1, Kerr method record ^218^Po counts for 10 min, while alpha spectrum method (method 5) and alpha/beta spectrum method (method 6, 7) only records the first 3 min counts for calculation. From this, we could deduce why Kerr's method gave a highest ^218^Po concentration than other methods. And we could understand that if more ^218^Po alpha counts (i.e., counts in the intervals following the first three 1 min interval) were used additionally to the first 3 min interval count, the other methods would give a ^218^Po values that maybe more precise and more close to Kerr's result. In order to prove this point further, we carried out another comparison. In this comparison, ^218^Po alpha counts of other 2 and 4 time intervals 3 min long were used along with the first 3 min count to calculate ^218^Po concentration. Taking the *α*_1_ *α*_2_(10) method as an example, the comparison results are shown in Table 3.

In Table 3, *α*_1_(d1 × 3 min), *α*_1_(d3 × 3 min), and *α*_1_(d5 × 3 min) mean using the first 3 min interval count, using the first three 3 min interval count, and using the first five 3 min interval count in ^218^Po concentration and EEC calculation. As shown in Table 3, the *α*_1_(d5 × 3 min) method gave larger Po-218 concentration than *α*_1_(d3 × 3 min) and *α*_1_(d1 × 3 min), which is close to the result of the Kerr method. And we could find that with the growing use of time intervals, such as the results of *α*_1_(d1 × 3 min) and *α*_1_(d3 × 3 min) and *α*_1_(d5 × 3 min), the Po-218 concentration will grow larger. From this point of view, the more time intervals, that is, the more counts, are used, the more precise ^218^Po is. Therefore, the Kerr method gives the best estimation of ^218^Po concentration.

## 6. Conclusion and Discussion

In this paper, a short review on radon progeny measurement methods was carried out, and then different measurement methods of radon progeny were compared both theoretically and experimentally. Those comparison results agree quite well and could lead to the following conclusion.

The spectrum method is more efficient than total counting method. The least-square calculation method is better than the resolved method in certain degree. Ten-interval method will give more precise PAEC than 8-interval method. The *αβ* spectrum method is more efficient than *α* spectrum method as well as nowadays' other measurement methods. However, for the measurement of ^218^Po concentration, the first and important progeny of radon, the Kerr method seems to be the best of all methods due to its 10 min record of *α*_1_ particle from ^218^Po.

Those comparisons also give us a lot of suggestion. The multiperiod counting of *α* plus *β* spectrum as well as using weighted least-square calculation method might be the best choice for accurate measurement of equilibrium equivalent concentration. More counts of ^218^Po should be recorded if we want to get precise concentration of ^218^Po, especially in the measurement of the unattached fraction of radon.

However, those suggestions do not mean we could easily use *αβ* spectrum analysis method to build a reference standard of radon progeny, because the detection efficiencies of *β* particles from radon progeny are hard to fix very precisely; that is probably the reason why PTB uses *αγ* spectrum method as standard method. As long as this problem can be solved, it is surely could be used as standard method. Considering this problem, Liquid Scintillation Spectrometry (LSC) might be a better choice.

## Figures and Tables

**Table 1:** Efficiency analysis of different radon progeny measuring methods.

	Methods	SD_PAEC_ (%)	Total time (min)	Efficiency index EI = 1/(SD ∗ Total time)	Sampling time (min)	Counting time (min)
1	Thomas 1972; *α*_1,2_	18.0	35	0.16	5	(7–10)(11–25)(26–35)
2	Nazaroff 1984; *α*_1,2_	6.3	60	0.26	5	(6–9)(12–29)(40–60)
		6.9	60	0.24		(7–10)(13–30)(42–60)
3	Kerr 1975; *α*_1_ *α*_2_	7.1	40	0.35	10	(12–22)(25–40)

4	*α*_1,2_(8)	16.2	35	0.18	10	(11–14)…(32–35)
	*α*_1,2_(10)	10.4	41	0.23		(11–14)…(38–41)
5	*α*_1_ *α*_2_(8)	8.1	35	0.35	10	(11–14)…(32–35)
	*α*_1_ *α*_2_(10)	6.1	41	0.40		(11–14)…(38–41)
6	*α*_1_ *α*_2_ *β*(1)	9.9	14	0.72	10	(11–14)
7	*α*_1_ *α*_2_ *β*(8)	3.4	35	0.84	10	(11–14)…(32–35)
	*α*_1_ *α*_2_ *β*(10)	3.1	41	0.78		(11–14)…(38–41)

**Table 2:** Experimental results of different methods.

Sampling time		Methods	EEC (Bq/m^3^)	Uncertainty (%)	Concentration (Bq/m^3^)		
					^ 218^Po	^ 214^Pb	^ 214^Bi
5 min	1	Thomas (*α*_12_)	3741	3.3	3226	3539	4158
	2	Nazaroff (*α*_12_)	3953	1.1	4151	4106	3690
			3988	1.2	4619	4197	3531

10 min	3	Kerr (*α*_1_ *α*_2_)	3970	1.2	6845	3495	3819
	4	*α*_12_(8)	3944	3.0	3028	3772	4431
		*α*_12_(10)	4002	1.9	3409	3912	4289
	5	*α*_1_ *α*_2_(8)	3915	1.4	5567	3687	3768
		*α*_1_ *α*_2_(10)	3908	1.0	5567	3668	3776
	6	*α*_1_ *α*_2_ *β*(1)	3979	1.1	5569	3988	3527
	7	*α*_1_ *α*_2_ *β*(8)	3996	0.4	5569	3977	3588
		*α*_1_ *α*_2_ *β*(10)	4090	0.4	5630	4097	3654

**Table 3:** Comparing Kerr's method with multiinterval method of increased *α*_1 _measurement.

	Kerr (*α*_1_ *α*_2_) *α*_1_(d1 × 10 min)	*α*_ 1_ *α*_ 2_(10)		
		*α*_ 1_(d1 × 3 min)	*α*_ 1_(d3 × 3 min)	*α*_ 1_(d5 × 3 min)
RaA (Bq/m^3^)	6845	5567	6158	6496
EEC (Bq/m^3^)	3970	3908	3937	3954
SD (PAEC) (%)	1.2	1.0	1.0	1.0
